# Clinical characteristics and treatment outcomes in patients with Niemann–Pick disease type C (NP-C): a cross-sectional study

**DOI:** 10.1186/s13023-025-03897-9

**Published:** 2025-08-27

**Authors:** Parvaneh Karimzadeh, Farzad Ahmadabadi, Vahide Zeinali, Sharareh Kamfar, Mahmoud Reza Ashrafi, Ali Reza Tavasoli, Seyed Hassan Tonekaboni, Faezeh Ghanaati

**Affiliations:** 1 Pediatric Neurology, Pediatric Neurology Research Center, Shahid Beheshti University of Medical Sciences, Tehran, Iran; 2 Research Institute for Children’s Health, Shahid Beheshti University of Medical Sciences, Tehran, Iran; 3 Pediatric Congenital Hematologic Disorders Research Center, Research Institute for Children’s Health, Shahid Beheshti University of Medical Sciences, Tehran, Iran; 4 Department of Pediatric Neurology, Pediatrics Center of Excellence, Children’s Medical Center, Tehran University of Medical Sciences, Tehran, Iran; 5 Pediatric Headache Program, Barrow Neurological Institute, Phoenix Children’s Hospital, Phoenix, AZ USA

**Keywords:** Niemann–Pick disease type C, Lysosomal storage disorder, Miglustat, Neurodegeneration, Treatment outcomes

## Abstract

**Background:**

Niemann–Pick disease type C (NP-C) is a rare autosomal recessive lysosomal storage disorder characterized by progressive neurodegeneration. This study aimed to characterize the clinical features and treatment outcomes of NP-C in Iranian patients.

**Methods:**

We conducted a cross-sectional study of 58 patients with NP-C diagnosed between March 2013 and March 2024 at Mofid Children’s Hospital, Tehran. Clinical manifestations were categorized into visceral, cortical, and deep brain domains. Treatment outcomes were assessed in 52 patients who received miglustat therapy.

**Results:**

The mean age at disease onset was 3.35 ± 3.40 years, with diagnosis occurring at 7.03 ± 4.60 years. Filipin staining confirmed diagnosis in 96.6% of cases. Following miglustat therapy (mean duration: 1.96 ± 2.54 years), 86.5% of patients remained stable or showed improvement in at least one disease domain. Visceral manifestations showed the most favorable response, with 30.8% of patients demonstrating improvement in hepatosplenomegaly. Cortical manifestations (seizures and cognitive disorders) improved in 13.5% of patients. Motor function remained stable in 63.5% of patients, while 7.7% experienced deterioration. Ocular manifestations remained largely stable (94.2%). Seven patients (13.5%) experienced deterioration in at least one domain. At study completion, mortality was 20.7%, with mean age at death of 11.58 ± 7.73 years.

**Conclusion:**

This first comprehensive analysis of NP-C in Iran demonstrates the effectiveness of miglustat across multiple disease manifestations, particularly for visceral and cortical symptoms. While neurological symptoms generally stabilized, responses varied, emphasizing the need for individualized therapeutic approaches. Early diagnosis and intervention remain crucial for improving outcomes in this progressive neurodegenerative disorder.

**Supplementary Information:**

The online version contains supplementary material available at 10.1186/s13023-025-03897-9.

## Introduction

Niemann–Pick disease type C (NP-C) is a ultra-rare autosomal recessive lysosomal storage disorder characterized by progressive neurodegeneration, hepatosplenomegaly, and premature mortality [1, 2]. The estimated incidence ranges from 1:100,000 to 1:150,000 live births, though this may be underestimated due to the challenging nature of diagnosis and variable age of onset [3–5]. The underlying pathophysiology of NP-C involves mutations in either the NPC1 gene (95% of cases) or the NPC2 gene (5% of cases), leading to impaired intracellular cholesterol trafficking and subsequent accumulation of unesterified cholesterol and glycosphingolipids in various tissues, particularly the brain [6].

The clinical presentation of NP-C is remarkably heterogeneous, with symptom onset ranging from the perinatal period to adulthood [7, 8]. While neurological involvement ultimately defines disease severity in most patients, initial presentations often include systemic manifestations such as cholestatic jaundice or isolated hepatosplenomegaly in infancy or early childhood [9]. The neurological manifestations exhibit age-dependent variation, spanning from developmental motor delays in early infancy to psychiatric disturbances in adult-onset cases [10]. Vertical supranuclear gaze palsy (VSGP) stands as the most characteristic neurological feature, serving as a crucial diagnostic marker [11].

Miglustat, an iminosugar that inhibits glucosylceramide synthase, represents the only approved disease-specific therapy for NP-C [2]. While several studies have demonstrated its effectiveness in stabilizing neurological manifestations [12–14], data on long-term outcomes and treatment response patterns in different ethnic populations remain limited. This knowledge gap is particularly pronounced in Middle Eastern populations, where genetic and environmental factors might influence disease progression and treatment outcomes. Population-specific genetic backgrounds, including potential founder effects or higher rates of consanguinity in Iran, may contribute to distinct mutation patterns, which could in turn affect disease severity, clinical manifestations, and even treatment tolerability compared to other population.

In Iran, despite advances in diagnostic capabilities and increasing awareness of NP-C, comprehensive data on the natural history of the disease and treatment outcomes remain scarce. Understanding the clinical spectrum, genetic profile, and treatment response patterns in this population is crucial for improving patient care and contributing to the global knowledge base of NP-C management. This study aimed to characterize the natural course of NP-C in Iranian patients and evaluate the effectiveness of miglustat therapy through an analysis of patients treated at a major referral center over a ten-year period.

## Method

### Study design and population

This cross-sectional study was conducted in accordance with the Strengthening the Reporting of Observational Studies in Epidemiology (STROBE) guidelines. We evaluated 58 patients diagnosed with Niemann–Pick disease type C (NP-C) between March 2013 and March 2024 at the Pediatric Neurology Department of Mofid Children's Hospital, Tehran, Iran. The diagnosis ofNP-C was based on a combination of clinical manifestations and confirmatory tests, including: (1) Filipin staining showing abnormal intracellular lipid storage. (2) Elevated LysoSM-509 biomarker levels. (3) Genetic sequencing analysis or reverse transcription polymerase chain reaction (RT-PCR). (4) Bone marrow aspiration (BMA) demonstrating typical foamy cells or sea-blue histiocytes. While the filipin staining test remains a valuable diagnostic tool in classic cases of NP-C, its invasive procedure, long turnaround time, and subjective interpretation limit its use in routine clinical practice. Therefore, we advocate for combining filipin testing with molecular genetic analysis whenever feasible to achieve a more definitive and reliable diagnosis (Supplementary Table 1). In this study clinical manifestations were categorized based on key neurological disease parameters introduced by Karimzadeh et al. [14] (Table 1).

**Table 1:** Key parameters of neurologic disease

Parameter	Grade	Manifestation
Ambulation	1	Normal gait
	2	Normal gait with minor loss of control (clumsiness)
	3	Ataxia
	4	Unable to walk
Fine and gross motor skills	1	Normal
	2	Tremor for object grasping and handwriting
	3	Unable to write and grasp objects
Swallowing	1	No difficulty in swallowing (normal)
	2	Mild dysphagia (only for solids)
	3	Moderate dysphagia (moderate difficulty for solids and minor dysphagia for liquids)
	4	Sever dysphagia (sever difficulty for solids and moderate difficulty for liquids)
	5	Unable to swallow
Speech	1	Normal speech
	2	Dysarthria
	3	Incomprehensive speech
Ocular movement	1	Slow ocular pursuit
	2	Vertical gaze palsy
	3	Vertical and horizontal gaze palsy
Hearing	–	Normal
	–	Abnormal
Seizure	–	No seizure
	–	Seizure controlled by antiepileptic drugs
	–	Refractory seizures

### Treatment and follow-up

As part of standard clinical care, patients without contraindications (e.g., pregnancy or severe disability) received miglustat therapy based on standard dosing protocols: 200 mg three times daily for adults, adjusted to 100 mg three times daily for infants based on body surface area. Clinical follow-up assessments were conducted at approximately six-month intervals. Each visit included a standardized neurological examination (assessing ambulation, speech, swallowing, and seizure activity), motor function assessment, and visceral evaluation. Visceral organ involvement, specifically hepatosplenomegaly, was assessed through both clinical examination and abdominal ultrasonography, which was performed at baseline and repeated annually or as clinically indicated. MRI of the brain was also conducted in selected patients to assess structural abnormalities.

Treatment response was determined by comparing clinical status at baseline (prior to initiation of miglustat) with the most recent follow-up. Outcomes in each symptom domain (neurological, visceral, motor) were categorized as improvement, stability, or deterioration, based on physician evaluation and documented patient/caregiver reports. Any enhancement compared to the patients’ prior status was classified as an improvement. All outcome data were extracted from structured clinic visit forms and electronic medical records.

In this study, “severe disability” refers to a combination of advanced motor impairment (e.g., inability to walk, severe muscle stiffness, and loss of coordination), cognitive decline (including loss of speech and memory), and functional dependency. Patients in this condition often experience swallowing difficulties, which increase the risk of choking or aspiration pneumonia, and they require full-time assistance with daily activities. In such cases, the decision not to initiate disease-specific treatment was based on the limited expected benefit and focus on palliative care to maintain comfort and quality of life.

### Statistical analysis

Statistical analysis was performed using SPSS version 27 (IBM, USA). Continuous variables were expressed as mean ± standard deviation or median with interquartile range, while categorical variables were presented as frequencies and percentages. Between-group comparisons were conducted using Pearson's Chi-square or Fisher's Exact test for categorical variables. Statistical significance was set at *p* < 0.05.

### Ethical considerations

The study protocol adhered to the ethical standards of the 1964 Declaration of Helsinki and received approval from the Ethics Committee of Shahid Beheshti University of Medical Sciences (IR.SBMU.REC.1395.29). Written informed consent was obtained from all patients or their legal guardians.

## Results

The study enrolled 58 patients with NP-C, with a male predominance (58.6%). The mean age at disease onset was 3.35 ± 3.40 years (range: 6 months-17 years), while the mean age at diagnosis was 7.03 ± 4.60 years (range: 1–23 years). Diagnostic confirmation through Filipin staining was positive in 96.6% of cases (56 patients), negative in 1.7% (1 patient), and not reported in 1.7% (1 patient). Baseline characteristics and clinical features of the study population are summarized in Table 2.

**Table 2:** Characteristics and clinical features of patients with NPC

Variables	
Sex^a^	
Male	34 (58.6)
Female	24 (41.4)
Age at onset (years)^b^	3.35 ± 3.40 (3, 0.5–17)
Age at diagnosis (years)^b^	7.03 ± 4.60 (6, 1–23)
Age at treatment (years)^b^	7.65 ± 7.12 (5.5, 0.5–35)
Filipin (*n* = 57)	
Positive	56 (98.2)
Negative	1 (1.8)
Brain MRI (*n* = 33)	
Normal	19 (58)
Cerebellar atrophy	7 (21)
Abnormal signal in periventricular white matter	5 (15)
Delay in myelination	1 (3)
Cortical and brain stem abnormality	1 (3)

^a^ = number (percent), ^b^ = mean ± standard deviation

Of the 58 patients, 52 received miglustat therapy for a mean duration of 1.96 ± 2.54 years. Six patients, being at end-stage disease, received no treatment. All 52 treated patients received therapeutic doses of miglustat. However, the dose of the drug was increased for 4 (7.2%) patients (Table 3). At therapeutic doses, juvenile patients received fixed doses of 200 mg three times daily, while infants were treated with body surface area–adjusted dosing (100 mg/m^2^, three times daily) in accordance with the manufacturer’s recommendations.. Patients with increased doses received ≥ 300 mg three times daily. Miglustat was generally well-tolerated. Mild, transient diarrhea occurred in 4 (7.2%) patients, resolving spontaneously in three patients with continued therapy. Abdominal discomfort was reported in only one case.

**Table 3:** Clinical manifestations of patient with leveled up miglustat dosage before and after the treatment

PID	Sex	Age at treatment	Visceral		Cortical		Ambulation		Fine and gross motor		Swallowing		Speech		Ocular	
			Pre	Post	Pre	Post	Pre	Post	Pre	Post	Pre	Post	Pre	Post	Pre	Post
P1	Male	2 years	–	–	Seizure	–	Ataxia	Ataxia	tremor	tremor	–	–	Dysarthria	Dysarthria	VSGP	VSGP
P2	Male	26 years	–	–	–	–	Ataxia	Ataxia	tremor	tremor	–	–	Dysarthria	Dysarthria	VSGP	VSGP
P3	Female	2.5 years	HSM moderate	HSM mild	–	–	Ataxia	Ataxia	tremor	tremor	–	–	Dysarthria	Dysarthria	VSGP	VSGP
P4	Male	7 years	–	–	–	–	Ataxia	Ataxia	tremor	tremor	–	–	Dysarthria	Dysarthria	VSGP	VSGP

Treatment response was evaluated across multiple clinical manifestations. Among patients with visceral manifestations (*n* = 23/52, 44.2%), post-treatment assessment showed stability in 7 patients (13.5%) and improvement in 16 patients (30.8%). Of the 21 patients (40.4%) presenting with cortical manifestations (seizures and cognitive disorders), 14 (26.9%) maintained stable condition and 7 (13.5%) demonstrated improvement following treatment.

Ambulation abnormalities were initially observed in 38 patients (73.1%). Following treatment, 33 patients (63.5%) maintained stable gait, while improvement and deterioration were observed in 1 (1.9%) and 4 (7.7%) patients, respectively. Fine and gross motor function abnormalities were present in 15 patients (28.8%); post-treatment evaluation revealed stability in 12 patients (23.1%), improvement in 2 (3.8%), and deterioration in 1 (1.9%).

Swallowing difficulties were initially present in 5 patients (9.6%), with post-treatment assessment showing stability in 3 patients (5.8%) and deterioration in 2 (3.8%). Speech abnormalities were documented in 25 patients (48.1%); following treatment, 21 patients (40.4%) remained stable, 3 (5.8%) showed improvement, and 1 (1.9%) experienced deterioration. All 52 patients presented with ocular manifestations at baseline; post-treatment evaluation demonstrated stability in 49 patients (94.2%), deterioration in 2 patients (3.8%), and improvement in 1 patient (1.9%). It is worth noting that in our study, ‘slow ocular pursuit’ encompassed both impaired smooth tracking and the characteristic vertical saccadic slowing observed in NPC patients, which is a well-recognized clinical sign of the disease.

Seven patients (13.5%) worsened in at least one of the manifestation categories. However, 45 patients (86.5%) were either stable in all manifestation categories or experienced improvement in some of them. Table 4 provides detailed information about patients'clinical manifestations before and after treatment.

**Table 4:** Clinical manifestations of patients with NPC before and after the treatment

Disease domains	Pre-treatment (*n* = 52)	Post-treatment (*n* = 52)	*P*-value
*Visceral manifestations*			< 0.001
No involvement	29 (55.80)	33 (63.50)	
HSM mild	7 (13.50)	16 (30.80)	
HSM moderate	15 (28.80)	2 (3.80)	
Splenomegaly	1 (1.90)	1 (1.90)	
*Cortical manifestations*			0.01
No involvement	31 (59.60)	38 (73.10)	
Seizure	6 (11.50)	0	
Cognitive impairment	15 (28.80)	14 (26.90)	
*Ambulation manifestations*			0.18
Normal gait	14 (26.90)	15 (28.80)	
Normal gait with minor loss of control	11 (21.20)	9 (17.30)	
Ataxia	23 (44.20)	21 (40.40)	
Unable to walk	4 (7.70)	7 (13.50)	
*Fine and gross motor manifestations*			0.56
Normal	37 (71.20)	39 (75.00)	
Tremor	14 (26.90)	11 (21.20)	
Unable to write	1 (1.90)	2 (3.80)	
*Swallowing manifestations*			0.22
Normal	48 (92.30)	47 (90.40)	
Mild dysphagia	2 (3.80)	2 (3.80)	
Moderate dysphagia	2 (3.80)	1 (1.90)	
Severe dysphagia	0	1 (1.90)	
Unable to swallow	0	1 (1.90)	
*Speech manifestations*			0.70
Normal	27 (51.90)	30 (57.70)	
Incomprehensive speech	10 (19.20)	6 (11.50)	
Dysarthria	12 (23.10)	12 (23.10)	
No talk	3 (5.80)	4 (7.70)	
*Ocular manifestations*			0.32
Slow ocular pursuit	26 (50.00)	25 (48.10)	
Vertical gaze palsy	26 (50.00)	27 (51.90)	

At the time of study completion, 12 (20.7%) patients had deceased, with a mean age at death of 11.58 ± 7.73 years (range: 2–26 years). Among them, seven deaths were directly attributed to complications of advanced NP-C. Two patients died from severe respiratory complications associated with COVID-19 infection. For the remaining three patients, the exact cause of death was not fully documented in the available records. However, based on clinical history and the absence of acute neurological decline, the deaths were presumed to be due to common complications observed in chronically ill pediatric patients. These cases were recorded as “presumed non-NP-C-related causes of death” due to the lack of specific confirmatory data.

## Discussion

This study provides a detailed characterization of NP-C among Iranian patients, contributing valuable insights into the clinical spectrum and treatment outcomes in this population. Our findings underscore the heterogeneity of NP-C and reinforce the role of miglustat in managing disease progression.

The clinical manifestations observed in our study align with the established phenotypic variability of NP-C. The mean age at onset (3.35 ± 3.40 years) and age at diagnosis (7.03 ± 4.60 years) are consistent with previous studies, highlighting diagnostic delays that remain a major challenge in NP-C management [15–17]. These delays primarily result from the broad clinical variability and heterogeneous presentation of the disease. Symptoms can range from neurological and psychiatric signs to systemic manifestations, making early diagnosis challenging [18]. In our study, Filipin staining confirmed the diagnosis in 96.6% of cases, consistent with international findings [6]. While genetic testing has enhanced diagnostic accuracy, limited access in resource-constrained settings contributes to diagnostic delays.

Brain MRI was performed in 33 out of 58 patients, of which 19 (58%) were reported as normal. Among the abnormal findings, cerebellar atrophy was the most frequent (7patients, 21%). These results differ from previous studies such as that of Lorenzoni et al., where diffuse brain and cerebellar atrophy were observed in approximately 60% of patients [19]. This disparity might reflect differences in disease stage at diagnosis or population-specific variations.

NP-C progressively worsens over time, and current treatment mainly focuses on stabilizing the patient's condition, delaying disease progression, and managing symptoms through supportive therapies. Miglustat remains the only approved disease-specific therapy for NP-C, with studies demonstrating its ability to stabilize neurological symptoms and delay disease progression [12]. In our study, 86.5% of patients receiving miglustat either maintained stability or showed improvement in at least one disease domain. While encouraging, these observations should be interpreted with caution as our study design does not allow for conclusions regarding causality.

Visceral manifestations appeared to improve in some patients following miglustat initiation, with 30.8% showing reduced hepatosplenomegaly. This finding supports previous reports suggesting that miglustat may provide systemic benefits in addition to neurological stabilization [14]. Similarly, cortical manifestations such as seizures were notably reduced post-treatment, reinforcing the drug's efficacy in mitigating epileptic events in NP-C patients [9]. Some patients in our study were concurrently receiving anti-epileptic medications and other supportive therapies, which could have contributed to the observed clinical benefits. Additionally, fluctuations in symptom severity are not uncommon in NP-C and may occur independently of disease-specific treatment. Therefore, attributing these changes directly to miglustat remains speculative without controlled comparisons.

In our study, motor function responses varied. While 63.5% of patients maintained stable ambulation, deterioration was observed in 7.7%. This progression is expected in NP-C due to its neurodegenerative nature, but the stabilization seen in most patients is encouraging. Swallowing and speech difficulties also showed mixed outcomes, with some patients experiencing deterioration, emphasizing the need for adjunctive supportive therapies [2].

VSGP was significantly present in our study, consistent with Lorenzoni et al.'s findings where VSGP served as a crucial diagnostic indicator [19]. This finding aligns with Patterson et al.'s observations, where neurological manifestations including ataxia and VSGP were prevalent in approximately two-thirds of patients [12].

At study completion, 20.7% of patients had deceased, with a mean age at death of 11.58 ± 7.73 years. This mortality rate aligns with international data, indicating that despite therapeutic advancements, NP-C remains a life-limiting disease with significant morbidity [1]. Early diagnosis and intervention remain crucial in improving long-term outcomes, particularly in regions where diagnostic resources are limited.

It is important to note that miglustat’s effectiveness can vary depending on the age at onset and individual patient characteristics [20, 21]. While some patients show significant improvements, others may not experience the same level of benefit. In our study, seven patients experienced deterioration in at least one disease domain. The mean interval between disease onset and medical intervention (miglustat) was approximately 3 years, highlighting the possibility that treatment response may be influenced by other characteristics such as the type and site of genetic mutations.

Our findings regarding miglustat’s effectiveness align with previous clinical observations, particularly in stabilizing neurological manifestations [21–23]. The high proportion of patients showing either stability or improvement across various symptom domains supports miglustat's role as the primary therapeutic option in NP-C management. Additionally, in four patients, dose escalation of miglustat was followed by observed improvements in clinical symptoms. This observation raises the possibility that higher doses of miglustat may be associated with better clinical outcomes, though further controlled studies are needed to confirm this.

In recent years, several novel therapies for NP-C have emerged, expanding the landscape of treatment options beyond miglustat. For instance, N-acetyl-L-leucine (NALL) has shown promising results in modulating lysosomal and metabolic dysfunction, with phase 3 trials demonstrating improvement in ataxia and quality of life. NALL has been reported to significantly reduce neurological signs and symptoms and appears to be safe and well tolerated [24]. Another agent, hydroxypropyl-β-cyclodextrin has shown potential in slowing neurological deterioration, although its effectiveness largely depends on the patient’s neurological status at the time of diagnosis. Importantly, disease progression is not fully halted, underscoring the need for early diagnosis and intervention, which could be facilitated by greater disease awareness and the implementation of newborn screening [25]. Furthermore, arimoclomol (Miplyffa), an oral medication recently approved in the United States for patients aged two years and older, has shown efficacy in attenuating neurological symptoms, particularly when used alongside miglustat [26]. However, these therapies were not available or accessible to our patients during the study period, and thus were not included in our treatment analysis.

This study provides the first comprehensive analysis of NP-C in Iran, contributing to the understanding of regional disease characteristics. The use of standardized diagnostic criteria and treatment protocols enhances the reliability of findings. However, limitations include the cross-sectional design, which restricts long-term outcome analysis, and the relatively small sample size, which may limit generalizability. Future longitudinal studies with larger cohorts are needed to further elucidate disease progression and treatment responses.

## Conclusion

This comprehensive analysis of 58 Iranian patients with NP-C demonstrates the effectiveness of miglustat therapy across multiple disease manifestations. Our findings highlight several key points: (1) early diagnosis remains crucial, (2) miglustat therapy shows particular effectiveness in stabilizing or improving visceral and cortical manifestations; (3) neurological symptoms, while generally stabilized by treatment, show variable responses, emphasizing the need for individualized therapeutic approaches; and (4) the drug demonstrates a favorable safety profile. This study underscores the importance of regular monitoring and individualized dose adjustment strategies. These findings contribute to the growing body of evidence supporting miglustat's effectiveness in NP-C management and provide valuable insights for clinicians treating this rare but severe condition. Future studies with longer follow-up periods and larger cohorts will be valuable in further understanding the long-term outcomes and optimal treatment strategies for NP-C patients.

## Supplementary Information

Additional file 1.

## Data Availability

The data supporting this study's findings are available on request from the corresponding author. However, the data are not publicly available because they contain information that could compromise the privacy of research participants.

## Abbreviations

NP-C: Niemann-Pick disease type C
VSGP: Vertical supranuclear gaze palsy
RT-PCR: Reverse transcription polymerase chain reaction
BMA: Bone marrow aspiration
SPSS: Statistical package for the social sciences
STROBE: Strengthening the reporting of observational studies in epidemiology
MRI: Magnetic resonance imaging
HSM: Hepatosplenomegaly
LysoSM-509: Lysosphingomyelin-509 (a biomarker for NP-C)
