# Supplementary material for: Clinical characteristics and treatment outcomes in patients with Niemann–Pick disease type C (NP-C): a cross-sectional study
Source: Orphanet J Rare Dis. 2025 Aug 27;20:459. doi: 10.1186/s13023-025-03897-9 (PMC12381998; doi:10.1186/s13023-025-03897-9)
Supplement: Supplementary file 1 — Additional file 1. [file 13023_2025_3897_MOESM1_ESM.docx]

Table 1. genetic finding of patients with NP-C

| Patient No | Gene | Nucleotide Change(s) | Protein change | Exon/Intron | Status |
| --- | --- | --- | --- | --- | --- |
| 42 | NPC1 | c.1534C > T | p. His512Tyr | Exon 9 | Homozygous |
| 44 | NPC1 | c.2776G > A | p. Ala926Thr | Exon 18 | Homozygous |
| 41 | NPC1 | c.3100G > A | p. Gly1034Arg | Exon21 | Homozygous |
| 27 | NPC1 | c.2974G > T | p. Gly992Trp | Exon 20 | Homozygous |
| 24 | NPC1 | c.2974G > T | p. Gly992Trp | Exon 20 | Homozygous |
| 61 | NPC1 | c.1433A>C | p. Asn478Thr | Exon 9 | Homozygous |
| 7 | NPC1 | c.3478-6T>A | - | Intron 22 | Homozygous |
|  | NPC2 | c.88G>A | p. Val30Met | Intron 22 | Homozygous |
| 16 | NPC1 | c.1990G>A | Val664Met | Exon 13 | Heterozygous |
|  |  | c.2821 T > C | p. Ser941Pro | Exon 13 | Heterozygous |
| 51 | NPC1 | c.1180 T>C | p. Tyr394His | Exon 8 | Homozygous |
| 56 | NPC1 | c.1415 T > C | p. Leu472Pro | Exon 9 | Homozygous |
| 55 | NPC1 | c.2086del | p. Ala696fs | Exon 13 | Heterozygous |
|  |  | c.1421C>T | p. Pro474Leu | Exon 9 | Heterozygous |
| 23 | NPC1 | c.1286T>G | p. Val429Gly | Exon 8 | Homozygous |
| 57 | NPC1 | c.1421C>T | p. Pro474Leu | Exon 9 | Heterozygous |
|  |  | c.428_429del | p. Glu143fs | Exon 4 | Heterozygous |
| 53 | NPC1 | c.2743A>T | p. Asn915Tyr | Exon 18 | Heterozygous |
|  |  | c.1831G>A | p. Asp611Asn | Exon 12 | Heterozygous |
| 30 | NPC1 | c.2821 T > C | p. Ser941Pro | Exon 19 | Heterozygous |
|  |  | c.2872C > G | p. Arg958Gly | Exon 19 | Heterozygous |
| 9 | NPC1 | c.1117G>C | p. Val 373Leu | Exon 8 | Homozygous |
| 6 | NPC1 | c.1433A>C | p. Asn478Thr | Exon 9 | Homozygous |
| 19 | NPC1 | c.3478-6T>A | p. Cys976Phefs*6 | Exon20 | Homozygous |
| 10 | NPC1 | c.1270C>G | p. Pro424Arg | Exon 8 | Homozygous |
| 8 | NPC1 | c.1180 T>C | p. Tyr394His | Exon 8 | Homozygous |
| 38 | NPC1 | c.1433A>C | p. Asn478Thr | Exon 9 | Homozygous |
| 1 | NPC1 | c.1433A>C | p. Asn478Thr | Exon 9 | Homozygous |
| 3 | NPC1 | c.1192C>T | p. His398Tyr | Exon 8 | Homozygous |
| 39 | NPC1 | c.1433A>C | p. Asn478Thr | Exon 9 | Homozygous |
| 4 | NPC1 | c.1192C>T | p. His398Tyr | Exon 8 | Homozygous |
| 5 | NPC1 | c.1970G>A | p. Gly657Asp | Exon 13 | Homozygous |
| 40 | NPC1 | c.1433A>C | p. Asn478Thr | Exon 9 | Homozygous |
| 18 | NPC1 | c.1166G>T | p. Arg389Leu | Exon 8 | Homozygous |
| 12 | NPC1 | c.2010C>G | p. Cys670Trp | Exon 13 | Homozygous |
| 13 | NPC1 | c.1970G>A | p. Gly657Asp | Exon 13 | Homozygous |
| 43 | NPC1 | c.2657dupG | p. Pro887Serfs*31 | Exon 18 | Homozygous |
| 21 | NPC1 | c.1970G >A | p. Gly657Asp | Exon 13 | Homozygous |
| 45 | NPC1 | c.2920_2923delCCTG | p.pro974profsTer8 | Exon 20 | Homozygous |
| 20 | NPC1 | c.2925-2928delCTGC | p. Cys976Phefs*6 | Exon 20 | Homozygous |
| 15 | NPC1 | c.2776G>A | p. Ala926Thr | Exon 18 | Homozygous |
| 34 | NPC1 | c.1970G>A | p. Gly657Asp | Exon 13 | Homozygous |
| 32 | NPC1 | c.506A>T | p. Asn169Ile | Exon 5 | Homozygous |
| 22 | NPC1 | c.1286T>G | p. Val429Gly | Exon 8 | Homozygous |
| 47 | NPC1 | c.2476_2484del | p. Ser826_Leu828del | Exon 16 | Homozygous |
| 46 | NPC1 | c.2476_2484del | p. Ser826_Leu828del | Exon 16 | Homozygous |
| 37 | NPC1 | c.2476_2484del | p. Ser826_Leu828del | Exon 16 | Homozygous |
| 33 | NPC1 | c.3732_3735del | p. Pro1245fs | Exon 24 | Homozygous |
| 59 | NPC1 | c.2010C>G | p. Cys670Trp | Exon 13 | Homozygous |
| 28 | NPC1 | c.3100G>A | p. Gly1034Arg | Exon21 | Homozygous |
| 31 | NPC1 | c.2776G>A | p. Ala926Thr | Exon 18 | Homozygous |
| 36 | NPC1 | c.2972_2973delAG | p. Gln991fs | Exon 20 | Homozygous |
| 29 | NPC1 | c.3100G>A | p. Gly1034Arg | Exon 21 | Homozygous |
| 35 | NPC1 | c.3100G>A | p. Gly1034Arg | Exon 21 | Homozygous |
| 2 | NPC1 | c.881+1G>T | - | splice donor variant | Homozygous |
| 52 | NPC1 | c.3100G>A | p. Gly1034Arg | Exon 21 | Homozygous |
|  |  | c.88G>A | p. Val30Met | Exon 21 | Homozygous |
| 58 | NPC1 | c.575A>G | p. Tyr192Cys | Exon5 | Homozygous |
| 17 | NPC1 | c.3100G>A | p. Gly1034Arg | Exon 21 | Homozygous |
| 54 | NPC1 | c.3282_3284del | p. Ile1095del | Exon 22 | Homozygous |
| 14 | NPC1 | c.2777C>T | p. Ala926Val | Exon 18 | Homozygous |
| 60 | NPC1 | c. 1421C>T | p. Pro474Leu | Exon 9 | Homozygous |
| 11 | NPC1 | c.1192C>T | p. His398Tyr | Exon 8 | Homozygous |
| 25 | NPC1 | c.1421C>T | p. Pro474Leu | Exon 9 | Heterozygous |
|  |  | c.428_429del | p. Glu143fs | Exon 4 | Heterozygous |
| 26 | NPC1 | c.1421C>T | p. Pro474Leu | Exon 9 | Heterozygous |
|  |  | c.428_429del | p. Glu143fs | Exon 4 | Heterozygous |
